# Cross Sectional Association between Spatially Measured Walking Bouts and Neighborhood Walkability

**DOI:** 10.3390/ijerph13040412

**Published:** 2016-04-08

**Authors:** Liang-Dar Hwang, Philip M. Hurvitz, Glen E. Duncan

**Affiliations:** 1 Interdisciplinary Graduate Program in Nutritional Sciences, University of Washington, Seattle, WA 98195, USA; liangdar@uw.edu; 2 Department of Urban Design and Planning, University of Washington, Seattle, WA 98195, USA; phurvitz@u.washington.edu; 3 Nutrition & Exercise Physiology Program, Washington State University, Spokane, WA 99210, USA

**Keywords:** Geographic Information Systems, residence characteristics, twins, walking

## Abstract

Walking is the most popular choice of aerobic physical activity to improve health among U.S. adults. Physical characteristics of the home neighborhood can facilitate or hinder walking. The purpose of this study was to quantify neighborhood walking, using objective methods and to examine the association between counts of walking bouts in the home neighborhood and neighborhood walkability. This was a cross-sectional study of 106 adults who wore accelerometers and GPS devices for two weeks. Walking was quantified within 1, 2, and 3 km Euclidean (straight-line) and network buffers around the geocoded home location. Walkability was estimated using a commercially available index. Walking bout counts increased with buffer size and were associated with walkability, regardless of buffer type or size (*p* < 0.001). Quantification of walking bouts within (and outside) of pre-defined neighborhood buffers of different sizes and types allowed for the specification of walking locations to better describe and elucidate walking behaviors. These data support the concept that neighborhood characteristics can influence walking among adults.

## 1. Introduction

In the U.S., walking is the most common form of physical activity, with national estimates indicating roughly 42% of adults walk during leisure time and 28% walk for transportation purposes [1] in intervals of at least 10 min. Regular participation in physical activity is beneficial for weight control and the prevention of chronic diseases [2,3]. Although efforts to increase levels of walking have received increased attention in recent years as an important means to improve population health [4], the majority of U.S. adults do not achieve levels of activity recommended for health while too many are completely sedentary [5,6]. Physical activity levels in the population are influenced by multiple factors ranging from biology to policy [7]. The role of the physical or “built” environment in supporting healthy lifestyles such as more walking for leisure and transportation has gained increased attention over the last decade.

The potential influence of neighborhood environment characteristics on walking levels, such as the presence of sidewalks, density of road network connections, and having utilitarian destinations within a short distance from the home is well documented [8] and further supported by findings that U.S. adults who live in urban areas have 4-fold greater walking levels (defined as walking trips of 1 mile or less) than those in rural areas [9]. The ability of a neighborhood to support walking and other forms of physical activity can be summarized with a neighborhood walkability index, which is typically based on measures of urban form such as street connectivity, land use mix, and residential density, as well as proximity to utilitarian destinations [10,11]. Several studies have shown that residents from highly-walkable neighborhoods walk more than those who live in less-walkable neighborhoods [12,13,14].

However, associations between walkability and walking levels commonly reported in the literature may be questioned for several reasons. First, many, but not all, studies have used self-report measures of walking rather than objective measures, which may have led to measurement error and bias. Second, the location of the walking activity is not typically specified in most studies, and so it is unknown whether the walking activity actually occurred within the neighborhood measured by the walkability index or in other distal locations. Third, the very definition of what constitutes a “neighborhood” is debatable; most descriptions of “walkable neighborhoods” are based on ease of walking to utilitarian destinations and urban form characteristics within pre-defined “buffers” around the home address (e.g., 400 m, 800 m and 1600 m, or roughly one-quarter to 1 mile around the home) [10,15]. As walkability is typically theoretically derived rather than empirically using objective data, whether these pre-defined buffers accurately capture objectively measured walking in areas around the home location (*i.e.*, neighborhood) is unknown.

To overcome the problems related to measuring walking activity noted above, we used methods to quantify objectively measured walking bouts as they occurred in space and time. The overall goal of this study was to quantify walking bouts that occurred within buffers of differing size (1, 2, and 3 km, or roughly one-half to 2 miles) and type (Euclidean, or “straight-line” *vs.* network-based buffers) around the home location, and to examine the association between walking episodes and neighborhood walkability. These cutoffs were selected because 1 km is a commonly accepted walking distance in the literature, [15] and walkability as estimated by Walk Score**^®^** (the index used in the present study, see methods for further description) best reflected the walkable amenities within a 1.6 km buffer [16], and falls between the 1 and 2 km buffers. The upper limit of 3 km is generally used in most studies that attempt to quantify spatial bouts of activity and walkable attributes [17,18]. We hypothesized that participants living in neighborhoods with higher walkability would have more walking episodes within their home neighborhood compared to those living in neighborhoods with lower walkability. Further, we investigated which buffer size and type best reflected walkability as quantified by a readily available walking index.

## 2. Experimental Section

### 2.1. Sample Characteristics

The subjects for this cross-sectional, secondary data analysis were sampled from a larger funded research project investigating the association between objective measures of physical activity and eating episodes with objective measures of urban form. Specifically, subjects in the present study were the first 106 individuals to complete the protocol of the parent study between June 2012 and October 2013, comprising 52 identical twin pairs and two individual twins. The parent study is ongoing and will ultimately include 200 identical, adult twin pairs (400 individuals) who were raised together but now reside separately within the Puget Sound region around Seattle, Washington. Twins are volunteers from the University of Washington Twin Registry (UWTR). Construction of the UWTR is described in detail elsewhere [19]. All participants completed a paper-based survey upon enrollment that included items on zygosity, sociodemographics (e.g., annual household income), height and weight, general health and common medical conditions, and lifestyle behaviors. Procedures for enrollment into both the twin registry in general, and the parent study from which subjects for the present secondary data analyses were obtained, were approved by the University of Washington Institutional Review Board (project identification code: 42315), with written consent obtained from each participant.

### 2.2. Measures

To measure daily physical activity, subjects were instructed to wear an ActiGraph GT3X+ triaxial activity monitor (ActiGraph, LLC, Pensacola, FL, USA) and a QStarz TR-Q1000XT GPS data logger (Qstarz International Co. Ltd., Taipei, Taiwan) attached to an elastic belt worn around the waist for 2 weeks. Accelerometer counts measured by the activity monitor provide estimates of the time spent in various intensity categories, including sedentary and light-, moderate-, and vigorous-intensity physical activity [20]. The accelerometer was configured to measure activity counts from each axis at 10-s epochs. Mean vector magnitude, calculated as $\sqrt{x^{2}+y^{2}+z^{2}}$, where *x*, *y*, and *z* represent the activity counts from each axis, was used as the measure of accelerometer counts in this study.

Latitude, longitude, and speed were recorded by the GPS device, also at 10-s intervals. The coordinates were used to locate the walking bouts in space, and speed was used in combination with accelerometer counts to exclude non-walking activity, using an algorithm described below.

The accelerometer and GPS data streams were integrated into 60-s epochs using common timestamps over the full 2-week period with ActiLife software (v. 6.8.1, ActiGraph, LLC, Pensacola, FL, USA), where GPS data were selected from the closest temporally matched measurement. Each record contained measures of date and time, accelerometer counts, latitude, longitude, and speed.

Neighborhood walkability was used as a measure of the built environment, estimated using the commercially available Walk Score**^®^** index [21]. Twin addresses were entered into the Walk Score**^®^** website, which uses data from business listings, road networks, schools, and public transit derived from multiple sources to map the walking distance to amenities in nine different categories (e.g., schools, parks, restaurants, *etc.*), with each category weighted by importance [22]. The algorithm then uses distances, counts, and weights to create a continuous score normalized on a scale of 0–100, with 0 representing the least (“Car-Dependent”) and 100 the most (“Walker’s Paradise”) walkable neighborhoods. This index has been used as a valid proxy of walkability for measuring access to walkable amenities in previous studies [16,23,24,25].

### 2.3. Data Processing

**Figure 1:**
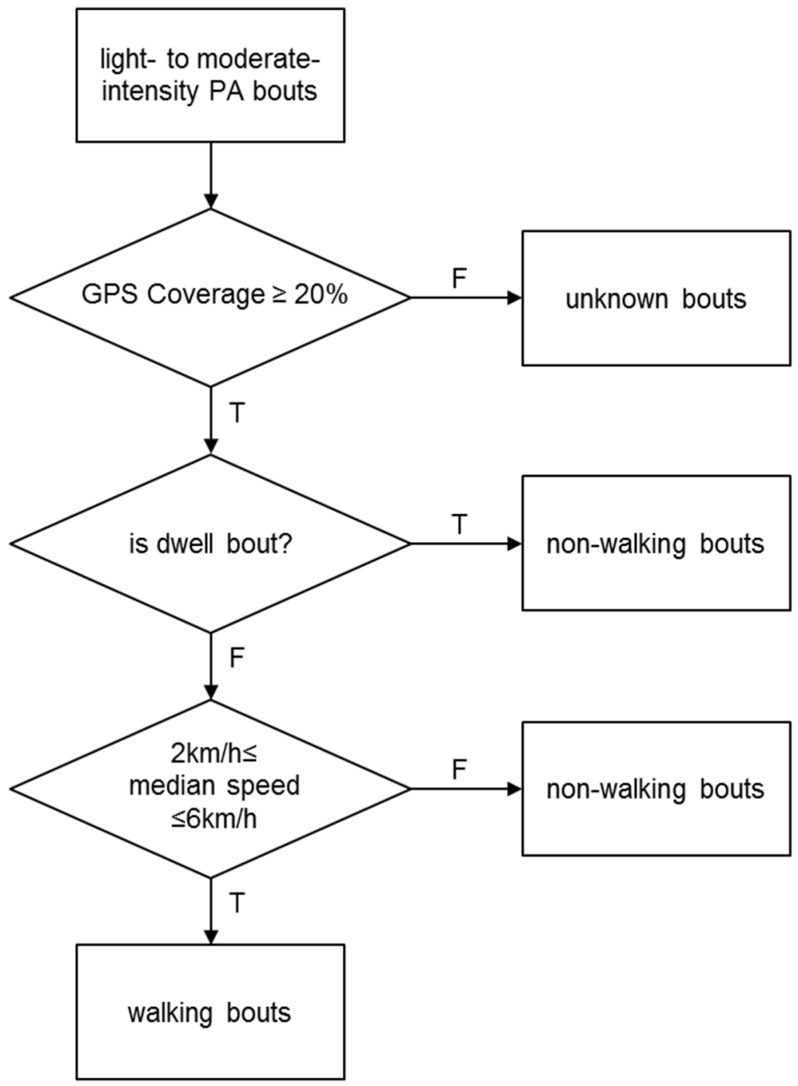
Decision-tree algorithm to classify physical activity (PA) into walking bouts.

Walking bouts were identified using a classification algorithm adapted from Kang *et al.* [26], depicted in Figure 1. Walking was defined *a priori* as non-mechanical and human-powered travel associated with sustained light- or moderate-intensity physical activity for at least 7 min in duration with a 2-min tolerance of lower physical activity intensity allowed within this interval [26]. Light- to moderate-intensity physical activity bouts were identified by accelerometer counts between 2000 and 6166 counts per minute epoch (cpe). Intervals having accelerometer counts >2000 cpe for at least 7 min, with up to 2 min below that threshold during the 7 min interval, were included as “preliminary” walking bouts. The threshold of 2000 cpe represents light-intensity physical activity at a speed of 3 km/h, indicative of walking, based on two studies that used the ActiGraph GT3X activity monitor to record the cpe during slow walking [27,28]. The upper bound of 6166 cpe corresponds to moderate-intensity physical activity at a speed of 6.4 km/h, is also indicative of walking [29]. Therefore, intervals with mean accelerometer counts >6166 cpe were considered non-walking bouts (*i.e.*, jogging or running).

Light- to moderate-intensity physical activity bouts identified using accelerometer counts of pre-defined speed ranges as noted above were subsequently considered walking bouts only when their corresponding GPS data satisfied three selection criteria. First, in order to provide sufficient spatial context information to distinguish between a walking bout and a non-walking bout, the bout required at least three GPS records, with ≥20% of bout records having matching GPS records. Second, bouts that occurred within a small spatial extent were considered as “dwells” and thus non-walking activities. To identify dwell bouts, the distances from each point to all other points within the bout were measured; bouts with their 95th percentile inter-point distance ≤40 m [30] were considered dwell bouts [26]. Only bouts with ≥10 GPS records were screened for being dwell bouts because non-dwell bouts with few GPS records were likely to have inter-point distance ≤40 m [26]. Finally, the GPS-derived median speed was required to range between 2 km/h and 6 km/h, based on two studies that identified walking trips in free-living conditions using GPS data [31,32].

**Figure 2:**
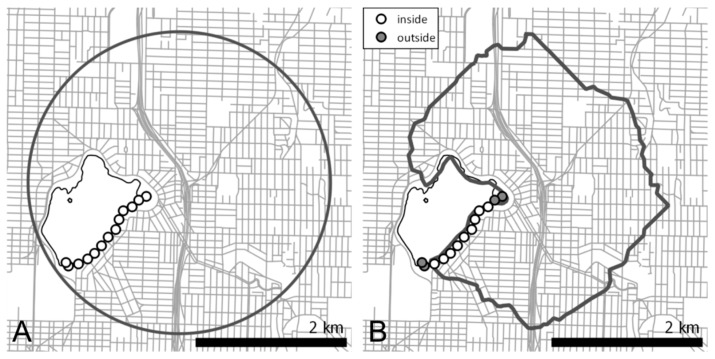
Two neighborhood buffer types drawn around the home location with resampled GPS points from a walking bout. (**A**) A walking bout entirely inside of a 2-km Euclidean buffer; (**B**) A walking bout partially inside and outside of a 2-km network buffer.

Two types of neighborhood buffers were created in ArcGIS 10.2 (Esri International LLC, Redlands, CA, USA). Figure 2A shows a Euclidean buffer formed as a circle around a geocoded address at a given radius. Home addresses from the UWTR were geocoded to parcel centroids using King County, WA address point GIS data for reference within ArcGIS 10.1 (Esri International LLC, Redlands, CA, USA). Figure 2B shows a network buffer, which is a polygon with edges defined by endpoints from all possible journeys an individual could travel from home to a given buffer distance. In comparison with Euclidean buffers, network buffers provide a more accurate estimation of the potentially accessible area for walking activity, because non-walkable areas such as water bodies and freeways are excluded [33]. Network buffers were created using street network data from the King County GIS Data Center using the ArcGIS Network Analyst extension. Both neighborhood buffer types were created using 1-, 2-, and 3-km radii around the home location, for the purpose of exploring possible effects of varying home neighborhood sizes.

Walking bouts were stratified for being within or outside the home neighborhood based on overlap with the home neighborhood buffers. Those bouts with all of their GPS points measured in the buffer were considered to have occurred within the home neighborhood (e.g., see Figure 2A using a Euclidean buffer), whereas bouts partially overlapping the buffer (e.g., see Figure 2B using a network buffer) were considered to occur outside of the home neighborhood buffer.

### 2.4. Statistical Analysis

Basic descriptive information was calculated as means and standard deviations (SD) or standard errors (SE), or percentages, where appropriate. The relationship between walkability and walking bouts was first examined using a Pearson’s correlation coefficient. Due to over-dispersed walking bout counts, a mixed-effects negative binomial regression model was used (Model 1) with walking bout counts as the outcome, the fixed factor of walkability (*WS*), and the random factor of twin pair ID. The random factor was used to account for the random effects between and within twin pairs because individual twins within a pair share genetic and common environmental experiences. Model 2 was adjusted for body mass index (BMI; calculated as weight in kilograms divided by height in meters squared), age, sex, and annual household income as a measure of socioeconomic status. To investigate which buffer size and type best reflected walkability, we used Model 2 to examine walking bout counts, within the three different neighborhood radii and two different buffer types, as a function of walkability.

All statistical analyses were conducted using R version 3.1.1 (R Core Team, Vienna, Austria). A *p*-value of 0.05 was used *a priori* to define statistical significance.

## 3. Results

The 106 participants aged between 24 and 70 years old (mean 41.7) with an average BMI of 27.4 kg/m^2^. More females than males were included in this study; 79% of participants had an annual income over $50,000. Roughly one-third each lived in “Car-Dependent” (0–49), “Somewhat Walkable” (50–69) and “Very Walkable” (70–89) categories, whereas 8.5% lived in the “Walker’s Paradise” (90–100) category. The overall mean Walk Score was 62.0 (Table 1).

A total of 4813 light- to moderate-intensity activity bouts were identified, spanning 1464 person-days across all 106 subjects, each of which had between 12 and 14 valid days of wear time. Of these activity bouts, 514 satisfied the GPS selection criteria and were classified as walking bouts (10.7%; Table 2). Non-walking bouts comprised 64% of all activity bouts, of which 18% were further classified as dwell bouts and 46% were out of range based on speed. Approximately 25% of the total activity bouts were classified as “unknown” due to a low GPS coverage ratio. The average duration of the walking bouts was 12.0 (SD 10.4) min. Participants had an average of 2.5 walking bouts per week, roughly equivalent to 30 total min of walking per week based on the average walking bout duration. Twenty subjects had no detected walking bouts over the entire 2-week measurement period.

**Figure 3:**
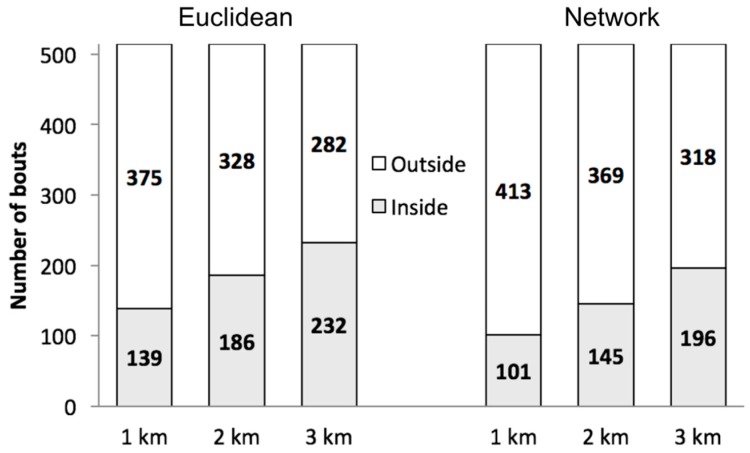
Total number of walking bouts within and outside of neighborhood buffers of different sizes and types for 106 subjects over two weeks of monitoring.

The counts of walking bouts are shown in Figure 3, with Euclidean buffers in the left panel, and network buffers in the right panel. More walking bouts were identified inside the neighborhood for Euclidean compared to network buffers of the same radius because Euclidean buffers cover a bigger area that completely includes network buffers (Figure 2). Irrespective of buffer type, the number of walking bouts inside the neighborhood increased with buffer size. More walking bouts occurred outside than inside of the neighborhood for all six buffers (e.g., 232 inside and 282 outside of the 3-km Euclidean neighborhood buffer), decreasing with buffer radius.

**Figure 4:**
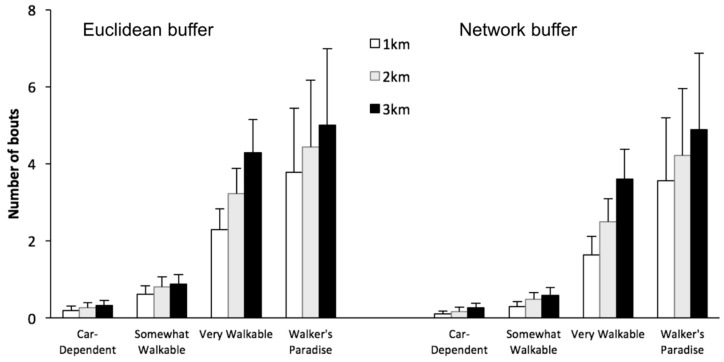
Average number of walking bouts inside buffers of different size and type over two weeks of monitoring, stratified by descriptive walkability categories.

The average count of walking bouts occurring in each neighborhood buffer increased monotonically for increasing buffer radii both within and across walkability levels, with the 3 km Walker’s Paradise category having the highest and the 1 km Car-Dependent category the lowest number of average walking bouts, respectively, for both Euclidean and network buffers (Figure 4).

The total number of walking bouts over two weeks was moderately associated with walkability (Pearson’s *r* = 0.39, *p* < 0.001). In Model 1, walkability was positively associated with the total number of walking bouts (*p* < 0.001) and the association remained significant after adjusting for age, sex, BMI, and income (Model 2), with every unit increase in WS associated with a 2.4% increase in walking bouts. A summary of regression model results is shown in Table 1. Walking was more prevalent for males and those with higher incomes, and less prevalent for older participants; there was no relationship with BMI.

Furthermore, Model 2 was used to examine the association between counts of all walking bouts within each pre-defined buffer and walkability. Table 3 shows that all associations were significant regardless of buffer size and type. The magnitudes of these regression coefficients did not statistically differ from each other (Table 3).

## 4. Discussion

The results of the present study provide new evidence for an association between neighborhood built environment characteristics and walking behaviors. The objectively measured walking bout counts were positively correlated with the neighborhood walkability score. These associations held when controlling for the correlated nature of the twin sample and for sex, age, BMI, and income. These covariate effects were generally comparable with current understanding [34], showing that men walked more than women and walking levels tended to decrease with age.

We further tested the walking-walkability association using a different set of buffer distances to define the neighborhood, quantifying walking episodes within 1-, 2-, and 3-km buffers of two different types, namely Euclidean and network-based. The associations between walkability and the number of walking bouts were all significant regardless of buffer size and type. Their regression coefficients were all higher than that calculated from the regression using the total number of walking bouts (non-stratified walking bouts). The results thus provide some additional insights into what constitutes a “walkable neighborhood”, suggesting that walkability (assessed using Walk Score**^®^**) reflects walkable destinations within neighborhood buffers ranging within the tested 1–3 km radii. To put these findings into perspective, an individual living in a “Walker’s Paradise” with a walkability value of 95 would perform about 400% more walking bouts inside a 2-km defined Euclidean neighborhood compared to an individual living in a “Car-Dependent” neighborhood with a walkability value of 25. In addition, the association pattern was much more pronounced in neighborhoods characterized as “Very Walkable” and “Walker’s Paradise” compared to “Car-Dependent” and “Somewhat Walkable” neighborhoods.

Dwell bouts were likely to have been activities performed within a small spatial extent such as walking on a treadmill or doing household chores such as gardening or vacuuming. Bouts with speeds out of walking range represented 45% of all physical activity bouts, most of which had a median speed below 2 km/h. These low speed bouts could potentially represent slow movements at a work place, such as walking between offices. Bouts with higher speeds could be biking or activities occurring in moving vehicles such as buses and trains. A further 1218 activity bouts were classified as “unknown” due to the low GPS coverage criteria. Missing GPS records could be due to lost signals in urban “canyons” or a device power outage [35,36]. While physical activity bouts with low GPS coverage were classified as unknown in the present study, Kang *et al.* [26] further classified bouts with incomplete GPS data using travel diaries, and approximately 50% were considered walking bouts. Thus, our number of 514 quantified walking bouts is likely low due to our strict objective methods, many of the 1218 unknown activity bouts would in turn likely be classified as walking with modified criteria or inclusion of ancillary data.

The average walking bout duration was 12 min, similar to that reported in previous studies [26,37]. The 1998 Behavioral Risk Factor Surveillance System reported a remarkably longer duration of 34.5 min per bout [38]; however, that study estimated only leisure-time physical activity *via* self-report, which was potentially subject to measurement error and recall bias. The frequency of walking in the present study was lower than that reported in the noted studies above, likely because the algorithm classified some walking bouts with low GPS coverage as unknown bouts.

We also found that, regardless of buffer size or type, the number of walking bouts inside was always lower than outside of the neighborhood. One explanation for this finding is that many walking episodes may have originated from a distal location rather than from home (or originated from home with a distal destination); we specifically did not restrict the starting point of each walking bout to the home location. For example, many of the walking episodes possibly originated at the work place, which was most likely outside of the neighborhood; however, work locations were not available for this study. There was no significant difference in the duration of walking bouts inside compared to outside of the neighborhood (data not shown), which may lend some support to our explanation that walking bouts outside of the neighborhood were less likely to have started from the home location, otherwise they would likely have been of a much longer duration. In addition, the number of walking bouts outside of the neighborhood was positively correlated with walkability (*r* = 0.12–0.28) regardless of buffer size or type. This suggests that participants who lived in neighborhoods with higher walkability not only walked more inside their neighborhood but also likely walked more outside of their neighborhood, compared to those living in neighborhoods with lower walkability. The consistent positive associations are supported by a recent study demonstrating that people who spend more time walking care more about walkable neighborhood attributes than those who engage in less walking [39]. This suggests that people who live in more walkable neighborhoods may walk more in general, both inside and outside of their home neighborhood.

Finally, when comparing the associations between walkability and the number of walking bouts inside each type of buffer, the regression coefficients were always slightly higher, though not significantly, for network buffers than those for Euclidean buffers of the same radius. This could be attributed to the fact that a network buffer represents a more realistic walking area because it considers street connectivity and excludes non-accessible locations. On the other hand, no obvious trend was observed for the buffer size, which seems compatible with a previous study showing subtle differences in the strength of associations across neighborhood size buffers (200- to 1600-m) [40].

There are some limitations worth noting in the present study. The algorithm failed to classify 25% of physical activity bouts due to low GPS coverage, resulting in many walking bouts being coded as “unknown” bouts. This issue could be resolved by using better locational technology that augments GPS signals with WiFi or cellular phone signals, or modified protocols that encourage subjects to check that their GPS devices are functioning normally throughout the data collection period. Although our analyses were restricted to walking bouts determined from objective measurements, the addition of travel diary data may have increased the number of bouts classified as walking or non-walking. We were unable to identify statistical differences in walking bout counts within the home neighborhood among different sizes and types of buffers. This is likely due to (1) the relatively low average number of detected walking bouts among our U.S. adult participants; and (2) a lack of statistical power despite a sample size over 100. Future analyses with bigger sample sizes could provide more precise results, as would using samples drawn from other populations that have higher overall levels of walking than the relatively sedentary U.S. population [5], and regions with more diverse levels of walkability than the relatively “car dependent” regions in the U.S. Walking behaviors may also have been influenced by geographical and climatic factors such as hills and inclement weather in the Seattle area. These factors may have significant influences on the walking levels measured in the present study, and should be considered when interpreting the strength of associations or generalizability to other regions. Lastly, although our study addressed many measurement issues (self-report data, the location of the walking activity and the size of the neighborhood), similar to the large body of neighborhood-effects studies published to date, we cannot establish causal associations between walkability and walking because of structural confounding, selection bias, and use of a cross sectional design. Our additional research [41] attempts to address these remaining critical issues by using innovative modeling techniques in a genetically informed sample of twins drawn from a community-based twin registry who have repeated measures of both exposures and outcomes.

## 5. Conclusions

The present study quantified walking bouts using objective accelerometry and GPS data without reliance on self-report surveys and travel diaries that have well established limitations. By quantifying walking episodes inside and outside of pre-defined neighborhood buffers of different sizes and types, we were able to provide some specification of the locations for walking that allowed us to better describe and elucidate walking behaviors in the context of participants’ physical surroundings. By using these methodological improvements, the results of the present study provide further evidence for an association between neighborhood environment features and walking behaviors among U.S. adults, as well as additional insights into the definition of what constitutes “walkable neighborhoods”.

## Figures and Tables

**Table 1:** Sample characteristics and association between walkability and walking bout counts identified over two weeks from 106 subjects.

Independent Variables		Mean (SD) or %	Model 1 *	*p*-Value	Model 2 *	*p*-Value
Walk Score		62.0 (22.5)	0.022 (0.005)	<0.001	0.025 (0.005)	<0.001
Sex (Male)		24.5%	-	-	0.643 (0.303)	0.034
Age		41.7 (10.5)	-	-	−0.015 (0.013)	0.237
BMI		27.4 (7.5)	-	-	0.001 (0.015)	0.930
Income	$19,999 and below	5.7%	-	-	-	-
	$20,000–49,999	14.3%	-	-	−0.553 (0.551)	0.315
	$50,000–79,999	26.7%	-	-	0.209 (0.485)	0.667
	$80,000 and above	53.3%	-	-	0.320 (0.489)	0.510

* Regression coefficients presented as betas with standard errors in parentheses.

**Table 2:** Description of physical activity bouts.

Activity Bout Classification	Counts	%	Duration in Minutes *
Walking	514	10.7	12.0 (10.4)
Non-walking			
Dwell	877	18.2	17.9 (10.1)
Speed out of range	2204	45.8	9.2 (7.4)
Unknown	1218	25.3	11.0 (8.1)
Total	4813	100	11.5 (9.1)

* Duration is shown as average minutes with standard deviations in parentheses.

**Table 3:** Association between walkability and walking bouts within buffers of different sizes and types. Covariates included sex, age, BMI, and income.

Buffer Type	Size	Beta *
Euclidean	1-km	0.054 (0.010)
	2-km	0.055 (0.009)
	3-km	0.050 (0.010)
Network-based	1-km	0.066 (0.013)
	2-km	0.061 (0.012)
	3-km	0.058 (0.010)

* Data presented as regression coefficients with standard errors in parentheses; *p* < 0.001 for all betas.
